# RELATIONSHIPS BETWEEN ANXIETY AND PERSONALITY DISORDER FEATURES IN YOUNGER AND OLDER ADULTS

**DOI:** 10.1093/geroni/igz038.1141

**Published:** 2019-11-08

**Authors:** Olivia R Noel, Lisa E Stone, Daniel L Segal

**Affiliations:** University of Colorado at Colorado Springs, Colorado Springs, Colorado, United States

## Abstract

Introduction: Anxiety is a prevalent problem that has been found to be associated with multiple other mental disorders, functional impairments, and poor quality of life. Specifically, it appears that personality may play a major role in anxiety based on preferred dispositional coping methods and presence of normal and dysfunctional personality traits. The purpose of this study was to examine associations between anxiety and personality disorder (PD) features. It was hypothesized that anxiety would have positive associations with the avoidant, dependent, obsessive-compulsive, schizotypal, paranoid, and borderline PD scales. Method: Community-dwelling older adults (N = 130) and younger adults (N = 243) completed the Geriatric Anxiety Scale (GAS) and the Coolidge Axis Two Inventory (CATI) as part of a larger assessment battery. Correlations were computed between the GAS total score and the 14 PD scales from the CATI. Results: Results showed that anxiety was significantly and positively associated with all 14 PD scales. Specifically, as expected, the schizotypal (.52), paranoid (.55), avoidant (.56), obsessive-compulsive (.60), dependent (.62), and borderline (.69) PD scales were all significantly positively associated with anxiety. The remaining 8 PD scales also showed strong, positive correlations with anxiety: sadistic (.27), antisocial (.28), schizoid (.32), histrionic (.42), narcissistic (.44), passive-aggressive (.59), self-defeating (.64), and depressive (.69). Discussion: These results indicate that anxiety and abnormal personality traits are highly associated, showing a strong comorbidity. An implication is that PDs may play a role in the development of anxiety, or vice versa. Longitudinal studies are needed to clarify temporal and causative relationships.

